# Two Decades of Insights: Comprehensive Histopathological and Epidemiological Analysis of Conjunctival Tumors

**DOI:** 10.3390/life14111381

**Published:** 2024-10-27

**Authors:** Dolika D. Vasović, Dejan M. Rašić, Zoran Latković, Bojana Dačić-Krnjaja, Jelena Vasilijević, Ivan Marjanović, Jelena Simonović, Anica Bobić Radovanović, Miodrag Karamarković, Milan Stojičić, Milica Mićović, Tanja Kalezić

**Affiliations:** 1 University Clinical Centre of Serbia, University Eye Hospital, Pasterova 2, 11000 Belgrade, Serbia; 2 Faculty of Medicine, University of Belgrade, 11000 Belgrade, Serbia; 3 School of Dental Medicine, University of Belgrade, 11000 Belgrade, Serbia; 4 Clinic for Burns, Plastic and Reconstructive Surgery, University Clinical Centre of Serbia, 11000 Belgrade, Serbia

**Keywords:** conjunctival tumors, histopathology, epidemiology, age distribution, tumor localization, gender distribution

## Abstract

This study analyzed 2102 conjunctival lesions excised between 1981 and 2003 at a single tertiary center in Serbia, with the aim of evaluating their histopathological characteristics, anatomical localization, and demographic distribution. Of the total cases recorded, 55.1% were male, indicating a slight male predominance. The bulbar conjunctiva was the most commonly affected site (34.5%), with 39.3% of tumors extended to multiple regions of the conjunctiva, including areas such as the plica and caruncula. The most common benign lesion was compound conjunctival nevus (16.7%), while squamous cell carcinoma (SCC) (11.4%) and melanoma (11.3%) were the most prevalent malignant tumors. Tumor incidence peaked in the 61–70 and 51–60 year age groups, with malignant tumors such as SCC being more frequent in males. Comparisons with similar global studies reveal that our findings align with worldwide trends, such as the predominance of SCC, which has been linked to UV exposure, and the frequency of melanoma in fair-skinned populations. However, the lower prevalence of fibrodegenerative lesions like pterygia and pinguecula in our cohort likely reflects Serbia’s cooler climate compared to regions with higher UV exposure. These findings underscore the diverse nature of conjunctival tumors, the critical role of histopathological examination for diagnosis, and the influence of environmental factors. This study provides valuable insights into the epidemiology of conjunctival tumors, contributing to global understanding and guiding future diagnostic and therapeutic approaches.

## 1. Introduction

Conjunctival tumors encompass a diverse group of lesions originating from the conjunctiva, the transparent mucous membrane that covers the sclera and lines the inner surfaces of the eyelids. These tumors exhibit a wide spectrum of clinical behaviors, ranging from benign lesions, such as conjunctival nevi and papillomas, to premalignant conditions, like conjunctival intraepithelial neoplasia (CIN), and malignant tumors, such as squamous cell carcinoma (SCC) and melanoma. While benign tumors are generally low-risk and often managed conservatively, malignant lesions pose significant clinical challenges due to their potential for local invasion, recurrence, and metastasis to regional lymph nodes or distant organs, which can greatly affect patient outcomes [1]. The proper classification and management of these lesions are therefore essential to optimizing treatment and prognosis. Histopathological evaluation remains fundamental in diagnosing and classifying conjunctival tumors, which are typically grouped based on their cellular origin. The most common benign tumors include melanocytic lesions, such as nevi, while non-melanocytic benign tumors are typically epithelial in origin, including squamous papilloma. Malignant tumors can be classified into melanocytic tumors, such as melanoma, and non-melanocytic tumors, such as SCC and lymphoma. Additionally, conjunctival tumors can be further categorized into primary, originating from the conjunctiva itself, or secondary, arising from adjacent tissues and invading the conjunctiva.

Conjunctival tumors account for approximately 2–5% of all ocular tumors globally, with significant geographic and demographic variation [1,2]. In regions with high ultraviolet (UV) exposure, such as Africa, Australia, and parts of South America, SCC is the most common malignant conjunctival tumor, reflecting the strong association between UV radiation and its pathogenesis [2]. Conversely, conjunctival melanoma, although rarer, is more frequently seen in fair-skinned populations, particularly in northern Europe and North America. Benign lesions, such as conjunctival nevi, are commonly observed across all populations, with a higher prevalence in younger individuals and Caucasians [2]. Conjunctival lymphomas, typically diagnosed in older adults, are increasingly linked to immunosuppression. These variations highlight the importance of region-specific studies to better understand the epidemiology of conjunctival tumors and lesions and the role of environmental and genetic factors in their development.

Several risk factors have been implicated in the development of conjunctival tumors, including ultraviolet (UV) radiation exposure, human papillomavirus (HPV) infection, and immunosuppression [2]. Additionally, genetic mutations, particularly BRAF mutations, have been linked to an increased risk of metastasis in conjunctival melanoma, further influencing diagnosis, prognosis, and treatment strategies [3]. A thorough understanding of these risk factors is crucial for early detection and intervention, especially in populations with high UV exposure or with a predisposition to genetic mutations.

Advances in diagnostic tools, such as slit-lamp biomicroscopy, immunohistochemistry, and refined histopathological techniques, have greatly improved the early detection and classification of conjunctival lesions, leading to more favorable clinical outcomes [2]. For instance, although SCC is relatively rare, it can be particularly aggressive, often necessitating a combination of treatments including surgical excision, cryotherapy, and adjuvant chemotherapy or radiotherapy to mitigate the risks of recurrence and metastasis [1]. Additionally, recent research has underscored the importance of genetic testing and the use of targeted therapies for conjunctival melanoma, which can significantly improve prognosis and guide personalized treatment plans [4]. Given the potential severity of malignant conjunctival tumors, timely diagnosis and intervention are essential to reducing morbidity and, in some cases, preventing mortality.

The purpose of this study was to analyze the spectrum of conjunctival lesions surgically treated over a 22-year period at a single tertiary center in Serbia, which serves as a referral center for the entire country and surrounding regions. This retrospective study is based on medical documentation from this clinic, offering a comprehensive overview of the histopathological characteristics of conjunctival tumors, as well as their distribution across different age groups and genders. By identifying trends in tumor types, anatomical localization, and demographic factors, this research provides valuable insights into the epidemiology of conjunctival lesions in this specific population. Furthermore, by comparing these findings with studies from other regions, we aim to contextualize our data within global patterns, offering a more comprehensive understanding of conjunctival tumor behavior and management.

## 2. Materials and Methods

This retrospective study analyzes conjunctival lesions surgically excised between 1981 and 2003, focusing on a broad spectrum of benign, premalignant, and malignant lesions. Data were collected from medical records at a single tertiary center in Serbia, including detailed demographic information such as age (categorized into decade intervals) and gender. Tumors were classified based on histopathological findings following excisional biopsy into benign (e.g., compound conjunctival nevi, hemangiomas, and squamous papillomas), premalignant (e.g., actinic keratosis and epithelial dysplasia), and malignant types (e.g., squamous cell carcinoma and melanoma). Tumor localization was meticulously documented, encompassing specific regions such as the bulbar conjunctiva, fornix, tarsal conjunctiva, limbus, caruncle, and semilunar plica, along with cases involving more than one of these regions.

Patients ranged from 0 to over 80 years of age, with their demographic data categorized by gender as male or female. Particular attention was given to the distribution of tumor types across these age groups and genders, as well as the anatomical site of tumor involvement. Surgical interventions were recorded, and histopathological classifications were used to confirm diagnoses. Cases with incomplete medical records or unclear diagnoses were excluded from the analysis to ensure the accuracy and reliability of the data. The final cohort provided a comprehensive overview of the types of conjunctival lesions encountered over the 22-year period.

The diagnosis for all cases was confirmed through histopathological examination based on excisional biopsy results. The study captured the most frequent tumor types, including SCC and melanoma, along with cases where tumors showed an extension of the lesion to several parts of the conjunctiva, including plica and caruncula. This detailed classification of tumor localization and histopathological type provided critical insights into the distribution patterns of conjunctival lesions in this population.

### Statistical Analysis

Descriptive statistics were employed to summarize the frequency, age distribution, gender distribution, and anatomical localization of the lesions. The chi-square test was used to assess associations between categorical variables, including lesion type by age group, gender, and localization. Statistical significance was defined as *p* < 0.05 for significant differences, while values of *p* < 0.001 were considered highly significant. All statistical analyses were performed using SPSS26.

## 3. Results

**Figure 1:**
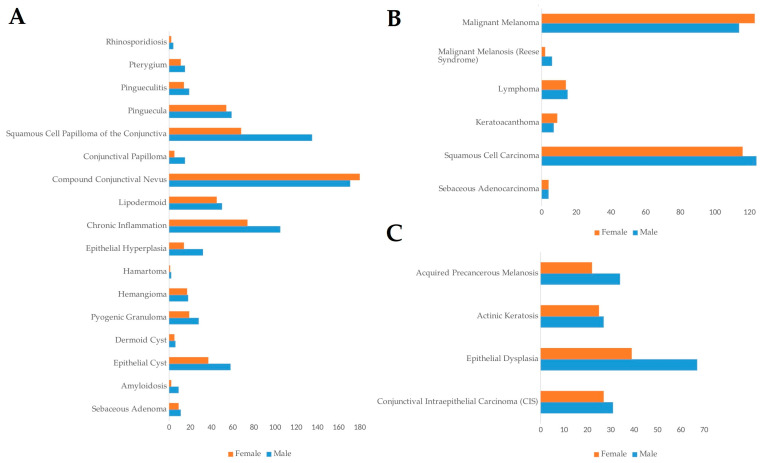
Frequency of conjunctival lesions based on gender. (**A**) Frequency in benign lesions; (**B**) malignant lesions; (**C**) premalignant lesions. All data are presented as the sum of cases.

This study analyzed 2102 conjunctival lesions excised between 1981 and 2003, with a focus on their frequency, anatomical localization, and demographic distribution. Of the total cases, 55.1% (1158 cases) were male, and 44.9% (944 cases) were female, indicating a slight male predominance in the occurrence of conjunctival lesions (Figure 1A–C). The mean age at presentation was concentrated in middle-aged adults, with the highest frequency observed in the 61–70-year age group (19.7%, 414 cases), followed by the 51–60-year age group (17.7%, 372 cases). This age distribution aligns with the known increase in tumor incidence with advancing age, particularly for both benign and malignant lesions.

**Figure 2:**
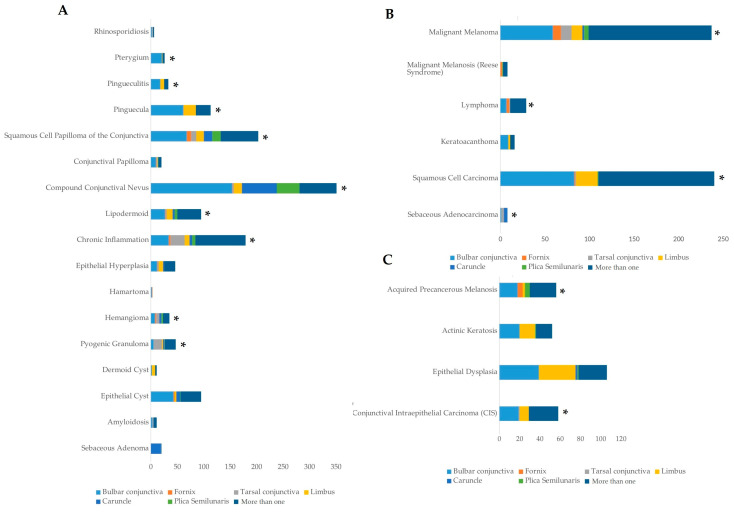
Frequency of conjunctival lesions based on tumor localization. (**A**) Frequency in benign lesions; (**B**) malignant lesions; (**C**) premalignant lesions. All data are presented as the sum of cases. An asterisk (*) indicates statistically significant difference (*p* < 0.001) in the distribution of lesions by anatomical location. The significance of the difference was estimated by the chi-square test.

In terms of anatomical localization, the bulbar conjunctiva was the most commonly affected site, accounting for 34.5% (725 cases) of the lesions (Figure 2). The extension of the lesion to more than one conjunctival region was prominent, observed in 39.3% of cases (827 cases). Other key sites of lesion localization included the limbus (9.5%, 200 cases), caruncle (6.3%, 133 cases), tarsal conjunctiva (4.2%, 89 cases), plica semilunaris (4.3%, 90 cases), and fornix (1.9%, 40 cases) (Figure 2).

**Figure 3:**
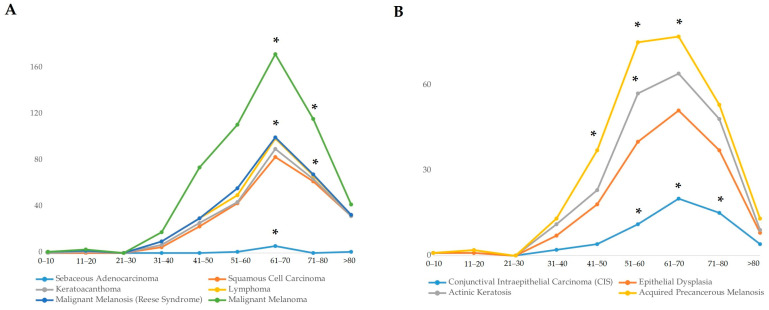
Age distribution of conjunctival lesions. (**A**) Malignant lesions; (**B**) premalignant lesions. All data are presented as the sum of cases. An asterisk (*) indicates statistically significant difference (*p* < 0.001) in the age of patients at the time of lesion detection. The significance of the difference was estimated by the chi-square test.

The most frequent benign lesion identified was compound conjunctival nevus, which accounted for 16.7% of all lesions (350 cases) (Figure 2A). Compound conjunctival nevus was significantly more common in younger patients (11–20 years, *p* < 0.05), indicating a predilection for benign tumors in younger individuals. SCC emerged as the most common malignant tumor, representing 11.4% (240 cases), closely followed by melanoma at 11.3% (237 cases) (Figure 2B). SCC showed a significant prevalence in the 61–70-year age group (*p* < 0.001), while melanoma also had a significantly high occurrence in patients aged 61–70 years (*p* < 0.001) (Figure 3A). Other notable tumor types included sebaceous adenocarcinoma (0.4%, 8 cases), conjunctival intraepithelial carcinoma (CIS) (2.8%, 58 cases), and squamous papilloma (9.6%, 203 cases) (Figure 2A–C). Sebaceous adenocarcinoma was more common in older patients, particularly those aged 61–70 (*p* < 0.001), while CIS was significantly present in the 51–80 age group (*p* < 0.001) (Figure 3A,B).

Regarding the gender distribution of the tumors, sebaceous adenocarcinoma had an equal distribution between males (4 cases) and females (4 cases), while SCC showed a slight male predominance with 51.7% of cases (124 cases) in males and 48.3% (116 cases) in females (Figure 1B). Melanoma exhibited a near-equal distribution, with 48.1% of cases (114 cases) in males and 51.9% (123 cases) in females (Figure 1B).

**Figure 4:**
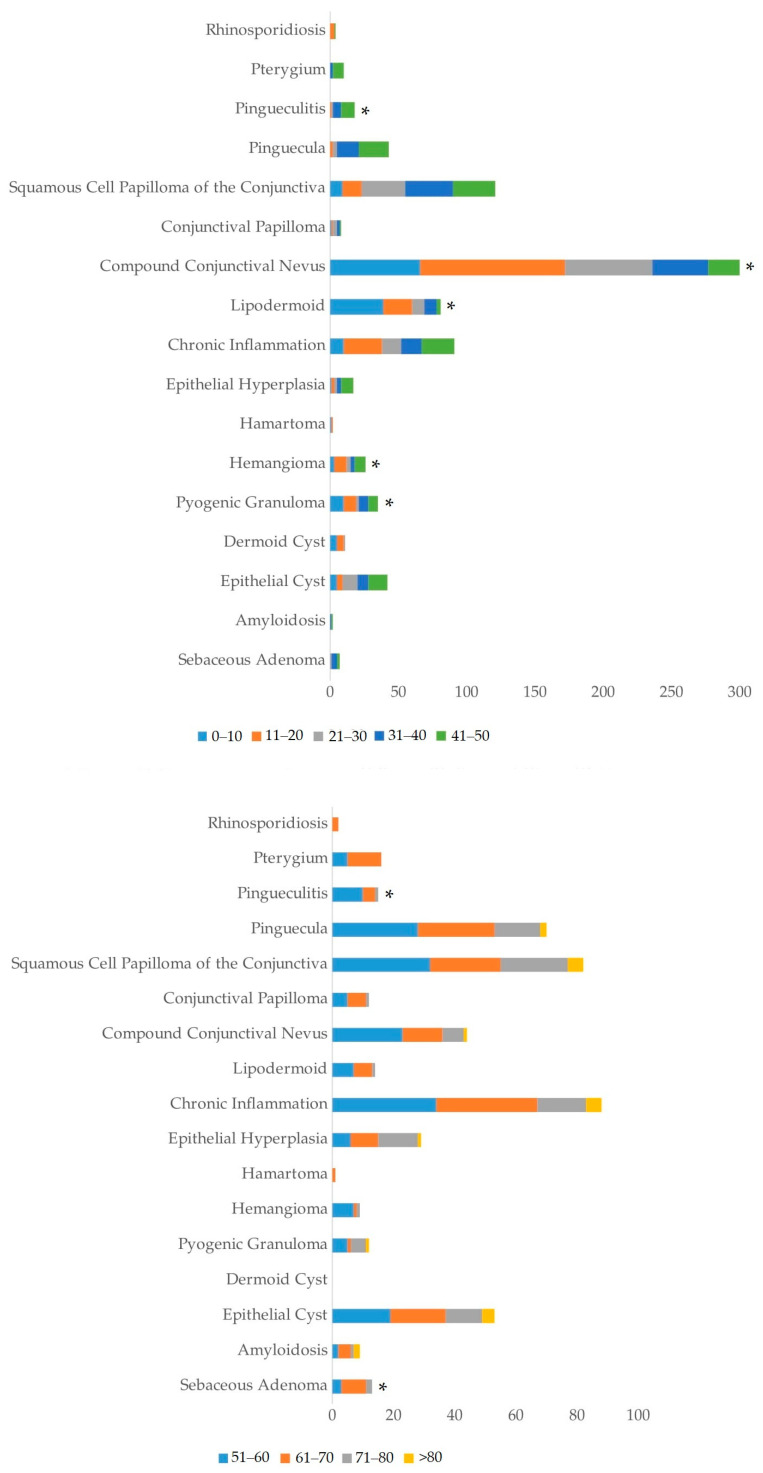
Age distribution of conjunctival lesions focusing on benign lesions. All data are presented as the sum of cases. An asterisk (*) indicates statistically significant difference (*p* < 0.001) in the age of patients at the time of lesion detection. The significance of the difference was estimated by the chi-square test.

The age distribution of different tumor types varied widely. Benign lesions such as compound conjunctival nevus were significantly more frequent in younger patients (11–20 years, *p* < 0.05), while malignant lesions like SCC and melanoma were more common in older patients, particularly those above the age of 50 (Figure 3 and Figure 4). SCC had the highest frequency in the 61–70-year age group (*p* < 0.001) and was also prevalent in the 71–80-year age group. Similarly, malignant melanoma was significantly more frequent in patients aged 61–70 years (*p* < 0.001) (Figure 3A).

## 4. Discussion

This research significantly contributes to our understanding of conjunctival lesions in a regional context and how they compare globally. One of the key objectives of this study was to assess the relative frequency of surgically treated conjunctival tumors, which are predominantly premalignant or malignant in nature, as benign lesions such as nevi are less commonly excised and may not be fully represented in these findings. SCC emerged as the most frequent malignant tumor in our cohort, representing 11.4% (240 cases) of all cases. This figure aligns with global trends. For instance, Shields et al. (2017) reported SCC accounting for 9% of conjunctival tumors in their large dataset, and Alkatan et al. (2022) found SCC to represent 50% of malignant conjunctival lesions in a 5-year study conducted in Saudi Arabia [5,6]. The consistency between our findings and other studies underscores the significant role of UV radiation as a risk factor for SCC development, especially in populations exposed to high levels of sunlight, which has been consistently supported by multiple studies [7,8]. SCC showed a significantly higher prevalence in the 61–70-year age group (*p* < 0.001), reflecting the impact of cumulative UV exposure (Figure 3A). This direct link between environmental UV exposure and SCC frequency is further emphasized by the prevalence of SCC in outdoor workers and populations living in sunny climates.

The frequency of melanoma in our study was 11.3% (237 cases), closely mirroring the 12% incidence observed by Shields et al. (2017) [5]. However, this result differs considerably from the findings of Alkatan et al. (2022), who did not report any cases of melanoma in their Saudi cohort [6]. This stark difference may reflect regional variations in UV exposure, genetic predispositions, and the prevalence of primary acquired melanosis (PAM), a known precursor to melanoma more frequently observed in fair-skinned populations [5,6]. Melanoma exhibited a significant prevalence in the 61–70-year age group (*p* < 0.001). In regions with lower UV exposure or different genetic backgrounds, the incidence of melanoma may be reduced, as seen in the Saudi cohort, where different environmental and genetic factors likely play a role in the decreased prevalence of melanoma. The occurrence of aggressive tumors, such as SCC and melanoma, highlights the critical importance of histopathological confirmation for these tumors, as both types are associated with significant morbidity and potential mortality if not treated early. The frequency of malignant lesions was significantly higher in patients over 50 years (*p* < 0.001), emphasizing the need for diligent follow-up and the thorough evaluation of any suspicious conjunctival growths.

Among benign tumors, conjunctival nevi were the most common lesions in our study, accounting for 16.7% of cases (350 cases). This result is comparable to the findings of Shields et al. (2017), who reported nevi in 23% of their cases [5]. However, our study contrasts sharply with findings from regions such as Turkey and Iran. For example, Findik (2019) in Turkey and Aliakbar-Navahi et al. (2015) in Iran reported a higher prevalence of fibrodegenerative lesions like pterygia and pinguecula, which comprised over 50% of benign lesions in their cohorts [9,10]. Compound conjunctival nevus was significantly more frequent in younger patients (*p* < 0.05), particularly in the 11–20-year age group. These discrepancies likely reflect regional differences in environmental conditions, particularly UV exposure. Pterygia and pinguecula are known to be associated with chronic UV exposure, which is more intense in regions with arid climates. In contrast, our Serbian cohort, exposed to a cooler climate, exhibited a lower incidence of these fibrodegenerative lesions, suggesting that the environmental factors of the region play a significant role in the type of benign lesions encountered. In addition to the already mentioned factors, the lower incidence of melanoma and conjunctival nevi in Saudi Arabia, Iran, and Turkey may also be influenced by lifestyle differences, including less frequent outdoor exposure, which could be due to climatic conditions and religious practices that limit time spent in open spaces.

Apart from the more common lesions such as SCC and melanoma, our study also identified several sporadic conjunctival lesions, including sebaceous adenocarcinoma, amyloidosis, and hamartoma. For instance, sebaceous adenocarcinoma was seen in only eight cases (0.4% of all lesions), a finding consistent with the rarity of the tumors also observed in other studies [11]. Amyloidosis was observed in 11 cases (0.5%), a rare entity that has been discussed in isolated reports, such as the case described by Ramberg et al. (2015), who reported sporadic cases of localized conjunctival amyloidosis [12]. Hamartoma, another infrequent lesion, was noted in three cases, consistent with isolated mentions in the literature [13]. These sporadic lesions, though rare, are clinically significant and emphasize the need for thorough histopathological examination even in uncommon cases [14].

Tumor localization was another key focus of our study. The excisional biopsies provided precise data on the anatomical distribution of conjunctival lesions. In our cohort, the bulbar conjunctiva was the most commonly affected site, with 34.5% of lesions (725 cases) arising in this region. This finding is consistent with the results of both Shields et al. (2017) and Alkatan et al. (2022), who also found the bulbar conjunctiva to be the most frequent site for both benign and malignant conjunctival tumors [5,6]. The bulbar conjunctiva’s direct exposure to environmental factors like UV radiation likely contributes to its frequent involvement, particularly in the development of SCC, a malignancy strongly associated with UV exposure [7]. Our data also revealed that 39.3% of tumors were located in more than one conjunctival region (*p* < 0.001). This is especially relevant for malignant tumors like SCC and melanoma, which are known for their invasive behavior and propensity to spread beyond the conjunctiva to adjacent ocular structures. Gichuhi et al. (2013) observed that advanced SCC often presents with lesions extending to multiple conjunctival regions, increasing the risk of recurrence and metastasis [8]. The significant frequency of tumor extension to multiple regions (*p* < 0.001) in our study underscores the importance of thorough examination and complete excisional biopsy to ensure all affected areas are adequately treated, particularly in malignant tumors with extensive spread. The gender distribution in our study revealed that 55.1% of conjunctival lesions occurred in males (1158 cases), consistent with other studies. Both Alkatan et al. (2022) and Shields et al. (2017) found a higher prevalence of conjunctival tumors in males, which may be attributed to lifestyle factors such as increased outdoor work and higher UV exposure in men [5,6]. A slightly higher male prevalence of malignant tumors like SCC (*p* < 0.001) may suggest underlying gender-related risk factors, which could include differences in exposure to environmental risk factors like UV radiation or genetic predispositions.

Age was another critical demographic factor in our study. The majority of lesions occurred in patients aged 50–70 years, which aligns with global trends. Older age has consistently been associated with an increased risk of both benign and malignant conjunctival lesions, as noted by Findik (2019) and Shields et al. (2017) [5,9]. SCC and melanoma were significantly more frequent in patients aged 61–70 years (*p* < 0.001). The cumulative effects of UV exposure over a lifetime likely contribute to the higher incidence of tumors in older individuals [5,9]. This age-related increase in conjunctival tumors, particularly malignant lesions like SCC, further emphasizes the importance of regular screenings and early detection in older populations. Sun et al. (1997) also emphasized that older patients have a higher likelihood of developing SCC due to prolonged UV exposure, further supporting the role of age as a significant risk factor [7].

Surgical excision remains the gold standard for managing both benign and malignant conjunctival lesions, as supported by several studies. Shields et al. (2017) and Alkatan et al. (2022) both stressed the importance of complete excision with clear margins, especially for malignant tumors like SCC and melanoma, which are prone to recurrence if not fully excised [5,6]. For patients with benign tumors like conjunctival nevi, the prognosis is excellent following surgical excision, with minimal risk of recurrence. However, malignant tumors like SCC and melanoma often required adjuvant therapies, such as cryotherapy and topical chemotherapy, to reduce the risk of recurrence. Gichuhi et al. (2013) and Ramberg et al. (2015) emphasized the importance of close postoperative monitoring for these malignant tumors, given their high recurrence rates and potential for metastasis [8,12]. Our findings of significant tumor extension to multiple conjunctival regions and older age as risk factors for recurrence (*p* < 0.001) further emphasize the need for diligent postoperative monitoring.

### Study Limitations

This study is based on data collected from a single tertiary center in Serbia between 1981 and 2003, which may limit the generalizability of the findings and does not account for more recent advancements in the diagnosis and treatment of conjunctival tumors. Furthermore, the study lacks detailed data regarding patients’ phototype, immune status, the presence of HPV infection, and other relevant risk factors, which may influence the development and progression of conjunctival tumors. While the study provides a comprehensive analysis of surgically treated cases, it is more representative of premalignant and malignant lesions, as benign tumors such as nevi were less likely to undergo excision during this period. Despite these limitations, the large sample size and long-term nature of the study offer a valuable foundation for future research in the field.

## 5. Conclusions

In conclusion, this study provides a detailed analysis of conjunctival lesions from a single center in Serbia, offering valuable insights into the frequency, localization, and histopathological characteristics of these lesions. When compared with studies from other regions, including Saudi Arabia, Turkey, and Egypt, our findings show both agreement and disagreement. For instance, the frequency of SCC and melanoma in our study aligns with global trends, but the lower prevalence of fibrodegenerative lesions like pterygia and pinguecula contrasts with findings from regions with more intense UV exposure. These differences underscore the influence of environmental factors on conjunctival tumor distribution. Future research should focus on integrating molecular markers and personalized treatment strategies to improve outcomes for patients with conjunctival tumors.

## Data Availability

Data are contained within the article.
